# Cardiovascular Mortality Associated with Low and High Temperatures: Determinants of Inter-Region Vulnerability in China

**DOI:** 10.3390/ijerph120605918

**Published:** 2015-05-27

**Authors:** Xunfeng Yang, Lianfa Li, Jinfeng Wang, Jixia Huang, Shijun Lu

**Affiliations:** 1 State Key Laboratory of Resources and Environmental Information System, Institute of Geographic Sciences and Natural Resources Research, Chinese Academy of Sciences, Datun Road, Beijing 100101, China; E-Mails: yxffcq@163.com (X.Y.); wangjf@lreis.ac.cn (J.W.); 2 University of Chinese Academy of Sciences, Beijing 100049, China; 3 Precision Forestry Key Laboratory of Beijing, Beijing Forestry University, Beijing 100083, China; E-Mail: jxhuang701@gmail.com; 4 College of Forestry, Beijing Forestry University, Beijing 100083, China; 5 National Institute for Nutrition and Health, Chinese Center for Disease Control and Prevention, 29 Nanwei Road, Xicheng District, Beijing 100050, China; E-Mail: lushijun666666@163.com

**Keywords:** cardiovascular mortality, temperature, socioeconomic and demographic factors, Poisson generalized additive model, Bayesian network

## Abstract

The objectives of this study were to estimate the effects of temperature on cardiovascular mortality in 26 regions in the south and west of China from 2008 to 2011, and to identify socioeconomic and demographic factors contributing to such inter-region variation in the temperature effect. A separate Poisson generalized additive model (GAM) was fitted to estimate percent changes in cardiovascular mortality at low and high temperatures on a daily basis for each region. The model used the smooth functions to model the nonlinear effects of temperature and humidity and to control for the seasonal factor using the calendar time variable. Given variation in the magnitude of the temperature effect on cardiovascular mortality, we employed a Bayesian network (BN) to identify potential region-specific socioeconomic and demographic factors that may explain the variation. In most regions, an increasing trend in high or low temperature was associated with an increase in cardiovascular mortality, with variation in the magnitude of the temperature effects across regions. Three factors, including *per capita* years of education (as an indicator of economic status), percentage of the population over 65 years of age and percentage of women had direct impact on cold-related cardiovascular mortality. Number of hospital beds (as an indicator of the availability of medical resources), percentage of population engaged in industrial occupations, and percentage of women showed direct impact on heat-related cardiovascular mortality. Due to the socioeconomic and demographic inequalities between regions, the development of customized prevention and adaptation programs to address the low/high temperatures in vulnerable regions should be prioritized.

## 1. Introduction

In the context of climate change, not only the global surface temperature but also the frequency, intensity, spatial extension and duration of extreme weather events have increased and will continue to increase [1]. In addition, many hospitalizations and deaths occur due to exposure to extreme weather conditions and climate events. The World Health Organization (WHO) estimated that climate changes, especially global warming, caused over 150,000 deaths and approximately five million “*disability-adjusted* *life years*” (DALYs) per year since the mid-1970s [2]. Models of the association between mortality and temperature are required to predict the consequences of global warming and dangerous weather conditions, develop efficient alert systems, and take preventive measures [3]. However, most studies of the effect of temperature on mortality have been conducted in developed countries. Though a few studies have been carried out in areas of China including Beijing [4], Shanghai [5], Tianjin [6], Guangzhou [7], and Hong Kong [8], there is still a lack of studies in multiple areas of China, which is the largest developing country with the largest population vulnerable to global warming, especially in less developed regions. Under the background of global warming, the annual mean temperature in China has increased by 0.45 ± 0.05 °C from 1860 to 2005 [9]. Heat wave duration and warm night show increasing trends in China, especially the Tibetan Plateau and Southwest China [10]. Meanwhile, increasing trends were also detected in the frequencies of warm days and warm nights [11], which have potential deleterious effect on human health.

Cardiovascular disease is the leading cause of deaths linked to climate fluctuations [12]. In general, the association between cardiovascular mortality and temperature displays a J-shaped [13], V-shaped [14] or U-shaped [15] pattern, indicating that both cold and hot temperatures are associated with an increase in cardiovascular mortality [16]. In the present study, we use a Poisson generalized additive model (GAM) to estimate the association between cardiovascular mortality and temperature on a daily basis for 26 regions in the south and west of China from 2008 to 2011. In general, the association between cardiovascular mortality and meteorological parameters is not necessarily linear [16], so we constructed the GAM to include time series variables using smooth functions to account for the nonlinear relationship between cardiovascular mortality and meteorological parameters [17].

In general, the magnitude of temperature effects on cardiovascular mortality is heterogeneous across regions due to variations in socioeconomic and demographic conditions [3]. Several previous studies have examined potential social factors responsible for regional vulnerability to temperature-related mortality [13,18]. However, the mutual and hierarchal relationships between the socioeconomic and demographic factors are complicated. This analysis proposed a Bayesian network (BN), which has the abilities to integrate multiple factors within a consistent system for risk assessment [19] and represent mutual and hierarchal relationships of variables [20], to explore region-specific socioeconomic and demographic factors responsible for the observed heterogeneity of the temperature’s effect on cardiovascular mortality. Moreover, the BN can offer insight into the impact of socioeconomic and demographic factors on the association between cardiovascular mortality and temperature, thereby providing relevant information for taking preventive steps and developing public health programs that target vulnerable populations in similar environments.

## 2. Materials and Methods

### 2.1. Study Area

**Figure 1:**
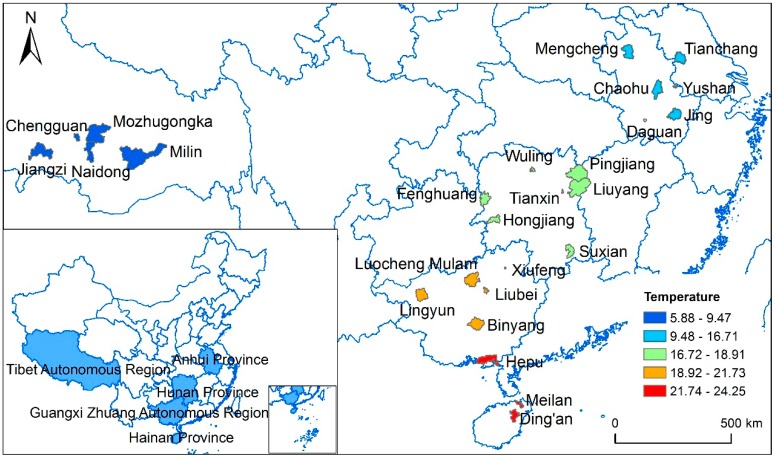
Spatial distribution of the 26 regions and the mean temperature during the study period.

This study was performed in 26 regions in the south and west of China (Table 1). Figure 1 shows the geographical distribution of the 26 regions. These regions are located between north latitudes 19°12′55″ N and 33°29′24″ N and east longitudes 89°8′8″ E and 119°13′35″ E. Hainan Province is located in a tropical region with hot summers and warm winters. Anhui Province, Hunan Province and Guangxi Zhuang Autonomous Region are located in a subtropical region with hot summers and cold winters. Xizang Autonomous Region is located in a plateau region with mild summers and cold winters.

**Table 1:** The 26 study regions.

Province	Region
Anhui Province	Chaohu City, Yushan District of Ma’anshan City, Daguan District of Anqing City, Tianchang City, Mengcheng County, and Jing County
Hunan Province	Tianxin District of Changsha City, Liuyang City, Pingjiang County, Wuling District of Changde City, Suxian District of Chenzhou City, Hongjiang City, and Fenghuang County
Guangxi Zhuang Autonomous Region	Binyang County, Liubei District of Liuzhou City, Xiufeng District of Guilin City, Hepu County, Lingyun County, and Luocheng Mulam Autonomous County
Hainan Province	Meilan District of Haikou City, and An’ding County
Tibet Autonomous Region	Chengguan District of Lasa City, Mozhugongka County, Naidong County, Jiangzi County, and Milin County

### 2.2. Source of the Data

The cardiovascular mortality data were extracted from the Nationwide Disease Surveillance Points (DSPs) System, and the regions were selected by stratified cluster random sampling by the Chinese Center for Disease Control and Prevention to carry out death surveillance in China. The completeness and accuracy of population enumeration in the DSPs has been evaluated using the standard United Nations Age Sex Accuracy Index. In our study, data on 26 DSPs covering 26 regions in the south and west of China from 2008 to 2011 were provided by the Chinese Center for Disease Control and Prevention. The original data provides population level data on causes of death, classification of disease (ICD-10), gender, age at death and date of death. We calculated the daily number of deaths from cardiovascular disease from 2008 to 2011 for each region. The International Classification of Diseases version 10 codes I00-I99 were used to define cardiovascular mortality. In our data set, cardiovascular diseases caused a total of 103,218 deaths.

Meteorological data from 2008 to 2011 for the study period, including daily mean temperature, daily maximum temperature, daily minimum temperature and daily mean relative humidity, were extracted from the China Meteorological Data Sharing Service System. We collected meteorological data from 674 meteorological monitoring stations covering the continent and Hainan Island of China. The inverse distance weighted (IDW) technique was used to interpolate the temperature and relative humidity data. Zonal estimation was used to aggregate the estimation of weather parameters falling into each cell of the target grid and calculate the mean temperature and mean relative humidity from the cells within the region polygon. The IDW technique and the zonal estimation were conducted using ArcGIS 10.1.

Demographic and socioeconomic information was extracted from the Tabulation on the 2010 Population Census of the People’s Republic of China by County [21], including the *per capita* years of education, the population percentage of uneducated people older than 15 years, the percentage of women, the percentage of urban residents, the percentage of the population older than 65 years, and the percentage of residents engaged in agricultural, industrial and service-related occupations. The number of hospital beds was extracted from the China Region Statistical Yearbook [22], China County Statistical Yearbook [23] and China Statistical Yearbook for Regional Economy [24].

### 2.3. Statistical Analysis

Our statistical analyses consisted of two stages. First, a separate Poisson GAM was fitted for each region to calculate the percent changes in cardiovascular mortality at cold and hot temperatures on a daily basis. Second, having observed variation in the magnitude of the temperature effect on cardiovascular mortality, a BN was employed to identify socioeconomic and demographic factors responsible for the variation in the cardiovascular mortality-temperature association.

#### 2.3.1. First Stage: Using a Poisson Generalized Additive Model (GAM) to Calculate the Percent Changes in Cardiovascular Mortality at Low and High Temperatures

There is broad agreement that both cold and hot temperatures are associated with an increase in cardiovascular mortality [13,14,15,25]. In this study, we used the 10th percentile of temperature as a reference value of low temperature and the 90th percentile as a reference value of high temperature [26,27,28]. The reference value of low temperature and high temperature for each region is shown in Supplementary Material (Table S1). Cardiovascular mortality depends not only on exposure to the current day’s temperature, but also on several previous days’ exposure [6], so we included the lagged effect by 2 weeks and 3 days for cold temperatures and hot temperatures respectively according to previous studies [14,15,16,29]. The following stratified lagged temperature variables were used for simplicity [16]: temperature (*T_1–3_*) over the preceding 3 days, temperature (*T_4–8_*) 4–8 days prior and temperature (*T_9–14_*) 9–14 days prior. Humidity was also incorporated in the model by means of smooth function. Finally, a nominal variable for day of the week was included to account for the week effect, and a smooth function of calendar time was included to account for some key potential effect modifiers, such as changes in population size, occurrence of major diseases, and potential seasonal confounders [13,16,30]. The final Poisson GAM for a given region had the following form [14,15,31,32,15,31]:

(1) $$\text{Log}\left(Y_{t}\right)=\alpha +\beta \left(T_{t}-\tau \right)+\overset{p}{\underset{j=1}{\sum }}s\left(x_{j},3\right)+s\left(time,7\times year\right)+\text{λ}\times DOW+{\text{ε}}_{t}$$

where *t* refers to the day of observation; *Y_t_* denotes the observed daily cardiovascular mortality from cardiovascular diseases on day *t*; *α* is the intercept term; *T_t_* denotes daily temperature on cold days or on hot days; τ is the reference value of low temperature or high temperature; β and λ are coefficients; *s*(.) represents a smooth relative risk function; *x_j_* denotes the covariates, such as relative humidity, with three degrees of freedom based on Akaike’s information criterion (AIC) [33]; *time_t_* denotes calendar date with seven degrees of freedom per year, a typical value chosen in these types of studies [15,16,34,35,36]; *DOW* represents the nominal factor for day of the week; and *ε_t_* is the residual. 

Models were constructed and compared considering different temperature indicators, namely mean temperature, maximum temperature and minimum temperature. Daily mean temperature stood out from the other two indicators based on AIC, so we used daily mean temperature as the indicator of temperature in our models.

The impact of low and high temperature was expressed as the percentage change in cardiovascular mortality for a temperature change of 1 °C [37]. The percent change was calculated using the following formula [36,38,39]:

(2) percent change=(exp(β)−1)×100

Cardiovascular mortality increases were evaluated by *percent changes*, specifically a 1°C increment above the high temperature threshold or a 1 °C decrement below the low temperature threshold. Poisson GAM analyses were conducted using the package mgcv [40] in R 3.0.1.

#### 2.3.2. Second Stage: Using a Bayesian Network (BN) to Identify Socioeconomic and Demographic Factors responsible for Variation of the Temperature Effect on Cardiovascular Mortality

Having observed variations in the magnitude of the effects of both low and high temperatures on cardiovascular mortality, we employed a BN to identify region-specific socioeconomic and demographic factors that may explain these variations. 

A BN represents a pair *B_S_* = (*G*, *P*), where *G* = (*V*, *E*) is a directed acyclic graph (DAG) over a finite set of random variables *V*, interconnected by directed links *E*, and *P* is a set of (conditional) probability distributions representing the conditional dependency relationships of the nodes. Each node representing a variable *A* with parent nodes representing variables *B_1_*, *B_2_*, ..., *B_n_* is assigned a conditional probability table (CPT) representing *P*(*A* | *B_1_*, *B_2_*, ..., *B_n_*) [19,20,41,42].

There are four steps required to construct a BN: feature selection, discretization, structure learning and parameter learning [19]. First, we selected the following 10 region-specific socioeconomic and demographic factors according to *a priori* knowledge and published literature: number of hospital beds per 10,000 population members [18]; *per capita* years of education; percentage of uneducated individuals among persons over 15 years of age (*%uneducated*) [18]; percentage of women (*%women*); percentage of urban residents (*%urban-residents*); percentage of the population over 65 years of age (*%65+*) [13]; percentage of residents engaged in agricultural (*%agriculture*) [18], industrial (*%industry*) or service-related (*%service*) [18] occupations; and climatic zone to account for the difference of physiological and behavioral patterns between different climatic zones.

Because a BN has a limited ability to address continuous variables, we had to discretize the factors before putting them into a BN. For discretization, either domain knowledge or a discretization algorithm can be used. If little domain knowledge of a variable was known, we referred to a discretization algorithm to make an automatic division. The report by Li *et al.* [19] provides details of this discretization method. The splits of the selected factors are shown Supplementary Material (Table S2). Structure learning is the process of learning the causal and influential dependence of the variables using a DAG. We employed a simulated annealing (SA) method to learn the structure of the BN [43]. SA is a generic probabilistic metaheuristic for the global optimization problem of locating a good approximation to the global optimum of a given function in a large search space. This approach randomly generates a candidate network close to the current network and accepts the network if it is better than the current one [43]. Parameter learning is the process of calculating the CPT for each variable based on the joint probability distribution of the network as follows [42]:

(3) $$P\left(\text{x}\right)=P\left(x_{1},\ldots ,x_{n}\right)=\overset{n}{\underset{i-1}{\prod }}P(x_{i}|pa\left(x_{i}\right))$$

where *P*(*x*) represents the joint probability distribution; *x* = *x*_1_,…, *x_n_* is a set of values of variables *X* = *X*_1_,…, *X_n_*; and *pa*(*x_i_*) is a set of values of the parents of *X_i_*.

We constructed a BN for cold-related and heat-related cardiovascular deaths, with the percent change in cardiovascular mortality at low temperatures and high temperatures as the target variable, respectively. The percent changes calculated in the first stage indicated that the heat-related cardiovascular mortality increased dramatically and cold-related cardiovascular mortality increased slightly, so we chose different reference values of “*high risk*” for cold-related and heat-related cardiovascular mortality. The target variables were classified as low risk or high risk using 1% as the threshold for cold-related cardiovascular mortality and 10% for heat-related cardiovascular mortality, respectively. That is, taking heat-related cardiovascular mortality for example, if the cardiovascular mortality increases by 10% or more with 1°C increment at high temperatures, the region will be classified as “*high risk*”. Consequently, 9 regions were classified as “*high risk*” at low temperatures, and 7 regions were classified as “*high risk*” at high temperatures.

Finally, we used a 2-fold cross-validation method to evaluate the performance of the BN. Three performance measures were employed: *pd*, *pf*, and *accuracy* [19]. *Pd* refers to the probability of detection and measures the probability of detection of a desired target. *Pf* refers to the probability of false alarms and measures the probability of undesired detections erroneously classified as targets. *Accuracy* measures the proportion of correctly predicted instances.

## 3. Results

In the Poisson GAM analysis, we calculated the percent changes in cardiovascular mortality at low and high temperatures for each region (see Supplementary Material Table S3). In most regions, cardiovascular mortality increased with increasing temperature on hot days and with decreasing temperature on cold days, with certain variations across regions. We used “0” as a reference value for the regions where an increasing or decreasing trend in the temperature on hot or cold days, respectively, did not have a deleterious effect on cardiovascular mortality.

**Figure 2:**
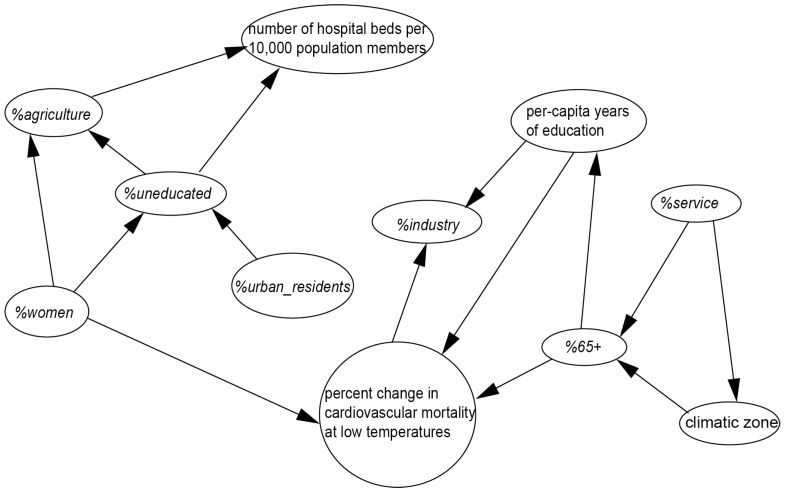
Bayesian network for percent change in cardiovascular mortality at low temperatures.

**Figure 3:**
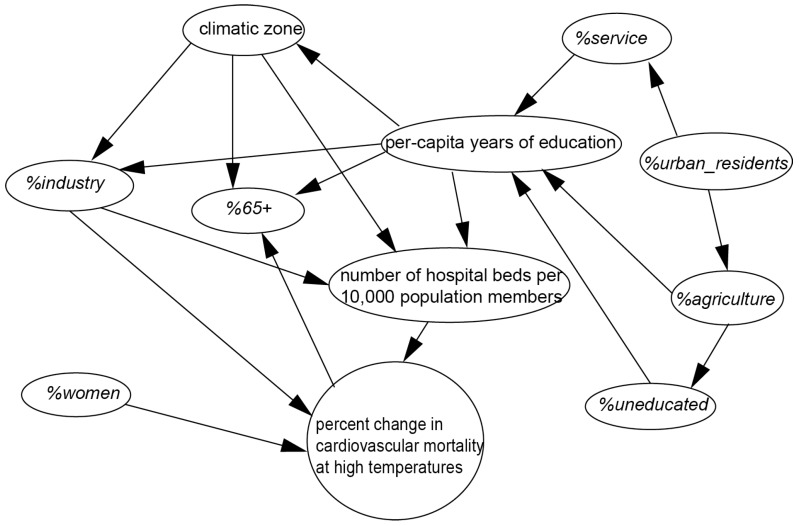
Bayesian network for percent change in cardiovascular mortality at high temperatures.

Figure 2 and Figure 3 show the modeled BNs for percent changes in cardiovascular mortality at low and high temperatures, respectively. Each elliptical node represents one socioeconomic or demographic factor, and the circular node represents the target variable. The arrows illustrate a probabilistic, causal or influential link from one node to another. Figure 2 shows that three factors, including the *per capita* years of education, the percentage of the population over 65 years of age, and the percentage of women had direct impact on the percent change in the occurrence of cardiovascular deaths at low temperatures. The marginal CPT (Table 2) shows that regions with a lower level of *per capita* years of education (less than 5.25 years) have a probability of 0.500 of being at high risk, which is higher than that with higher level of *per capita* years of education (more than 5.25 years) with a probability of 0.342. Regions with less old population (less than 5.65% with a probability of 0.690) have a higher probability of being at high risk than that with more old population (more than 5.65% with a probability of 0.273). Regions with less women (less than 50.08% with a probability of 0.381) have a higher probability of being at high risk than that with more women (more than 50.08% with a probability of 0.209).

Figure 3 shows that three factors, including number of hospital beds per 10,000 population, the percentage of residents engaged in industrial occupations, and the percentage of women had direct impact on the percent change in cardiovascular deaths at high temperatures.

**Table 2:** Marginal conditional probability table (CPT) of percent change in cardiovascular mortality at low temperatures.

Quantitative Factors	State and Intervals	Percent Change in Cardiovascular Mortality at Low Temperatures	
		Low Risk	High Risk
*Per capita* years of education	low level [5.18, 5.25]	0.500	0.500
	high level (5.25, 12.31]	0.658	0.342
%*65+*	low level [3.49, 5.65]	0.310	0.690
	high level (5.65, 12.68]	0.727	0.273
%*women*	low level [46.66, 50.08]	0.619	0.381
	high level (50.08, 50.94]	0.791	0.209

Furthermore, the CPT (Table 3) shows that regions with less hospital beds (30.71 per 10,000 population) have a probability of 0.599 of being at high risk, which is higher than that with more hospital beds (more than 30.71 per 10,000 population) with a probability of 0.181. And regions with more women (more than 50.83%) have a higher probability (0.546) of being at high risk than that with less women (less than 50.83% with a probability of 0.479). The factor of the percentage of residents engaged in industrial occupations was discretized into three intervals by the automatic discretization algorithm, with the low level (less than 6.36% with a probability of 0.805) associated with the highest probability of being at high risk, followed by the high level (more than 23.14% with a probability of 0.297), and the middle level (between 6.36% and 23.14% with a probability of 0.130) associated with the lowest risk.

**Table 3:** Marginal conditional probability table (CPT) of percent change in cardiovascular mortality at high temperatures.

Quantitative Factors	State and Intervals	Percent Change in Cardiovascular Mortality at High Temperatures	
		Low Risk	High Risk
Number of hospital beds per 10,000 people	low level [4.95, 30.71]	0.401	0.599
	high level (30.71, 109.38]	0.819	0.181
%*industry*	low level [1.02, 6.36]	0.195	0.805
	middle level (6.36, 23.14]	0.870	0.130
	high level (23.14, 51.24)	0.703	0.297
%*women*	low level [46.66, 50.83]	0.521	0.479
	high level (50.83, 50.94]	0.454	0.546

Finally, Table 4 shows the results of a 2-fold cross-validation of the modeled BNs.

**Table 4:** 2-fold cross-validation of the BNs.

Target Variables	State	pd	pf	Accuracy
Percent change in cold-related cardiovascular mortality	low risk	0.882	0.333	0.808
	high risk	0.667	0.118	0.808
Percent change in heat-related cardiovascular mortality	low risk	0.947	0.143	0.923
	high risk	0.857	0.063	0.923

## 4. Discussion

The objectives of this research were to characterize the association between temperature and cardiovascular mortality in 26 regions in the south and west of China and to identify the influence of socioeconomic and demographic factors on this association in different regions. Several previous studies have examined potential social factors responsible for regional vulnerability to temperature-related mortality. Curriero *et al.* used a linear regression model to study the impact of social economic and demographic factors on cold-related and heat-related cardiovascular mortality [13]. They regressed cold-related (or heat-related) cardiovascular mortality on each predictor alone as social-economic and demographic factors are generally correlated, which may lead to a problem with multicollinearity. The study of Curriero *et al.* had the limitation of not able to model the intercorrelation of the factors. To deal with multicollinearity, Wu *et al.* used principal component analysis (PCA) to classify the demographic and socio-economic factors into four integrated factors [18]. However, the social factors extracted using PCA were not realistic, which made it difficult to reflect realistic problems or to provide useful suggestions for public health specialists. In contrast with previous studies, this analysis proposed a BN to integrate multiple factors within a consistent system, which has the ability of modeling the mutual and hierarchal relationships of the variables without causing a problem of multicollinearity, thereby offering a comprehensive understanding of the relationships between the factors and temperature-related cardiovascular mortality. Our results suggest that a BN is a good alternative for risk assessment in public health.

In the BN analysis of the percent changes in cold-related cardiovascular mortality, three factors including *per capita* years of education, the percentage of the population over 65 years of age and the percentage of women were identified as influential factors. Education attainment is a robust indicator of socioeconomic position, income, living conditions and occupation of the residents of a region [44]. In general, persons with high education attainment have an improved quality of life [44] and use heating equipment in their living and working environments. However, the less educated population may have limited access to medical resources, poor housing conditions, a lack of necessary health knowledge and unhealthy behavioral habits such as smoking and drinking. In the present study, higher risk for the younger people at cold temperatures was observed. Although several studies have reported that elderly people are more sensitive to high/low temperatures, some studies have observed stronger effects in younger subjects [45,46,47]. One possible explanation might be that the elderly group includes more people who are often confined to beds or home and are thus less exposed to cold temperatures. In contrast, younger people tend to work outside and thus may be directly exposed to ambient temperatures, which makes them more influenced by cold temperatures [45].

In the BN analysis of the percent changes in cardiovascular mortality at high temperatures, 3 factors including number of hospital beds, the percentage of residents engaged in industrial occupations and the percentage of women showed an obvious influence [18,48]. The number of hospital beds may serve as an indicator of medical resources, and adequate medical resources may have a protective effect against high temperatures in cardiovascular patients. In addition, air conditioning units are often available in nursing homes or hospitals, and people nursed in a hospital are more likely to receive adequate medication and nursing care and less exposure to heat [27].

The influence of industrialization is complicated, with the low level associated with the highest risk, followed by the high level, and the middle level associated with the lowest risk. The industrial sector usually contributes to air pollution, which may have deleterious effects on public health. But a high level of industrialization also typically corresponds to improved economic status, convenient transportation, a controllable indoor working environment, and good medical infrastructure. The findings in this study led us to hypothesize that the reason why the low level of industrialization is associated with highest risk is mainly because of the low economic status. As the industrialization develops, the beneficial impact of developed economy is more pronounced than the deleterious impact of air pollution. But after a certain development level, the deleterious impact of air pollution exceeds the beneficial impact of developed economy. Nevertheless, the impact of the industrial sector on cardiovascular disease and mortality is complex, and few studies have been performed to evaluate this issue.

Gender showed different impact at low and high temperatures. Men tend to have higher risk at cold temperatures, while women tend to have higher risk at high temperatures. The mechanism remains unclear. It may be that women usually have more fat than men, which makes women more cold-resistant but less heat-resistant. The difference of the temperature effect on mortality between women and men was dependent on study location and study population. Some previous studies have reported that women show higher risks than men in Korea [49] and Mexico City [50], while men were more severely affected in São Paulo [50]. The influence of gender is geographically diverse, so location-specific assessment is needed when developing location-specific public health programs.

During cold or hot temperature events, people with cardiovascular disease represent a vulnerable subgroup, and various hypotheses have been proposed to explain the mechanism responsible for the adverse effect of cold and hot temperatures on cardiovascular patients. In hot weather, the body tends to enlarge skin vessels and increase sweating to balance the distribution of heat, thus inducing profound physiological changes such as an increase in blood viscosity and cardiac output. These changes lead to dehydration, hypotension, and even endothelial cell damage [14,25,51]. However, less is known about cold-related mortality [25,46]. One plausible hypothesis suggests that when exposed to cold temperatures, the sympathetic nervous system increases the catecholamine level and the skin vessels then constrict to reduce heat loss, thus inducing vasoconstriction and increases in blood pressure, sympathetic nervous activities, platelet aggregation, red blood cell count, plasma cholesterol and plasma fibrinogen [14,25,52].

Some limitations exist in the present study. First, some studies have reported that air pollutants might be potential confounders and/or effect modifiers of the mortality-temperature association [3,25,53], but we don’t adjust our models for air pollution due to lack of data. In many locations, concentrations of pollutants may be associated with temperature [3]. Previous studies on the associations between air pollutants, temperature and mortality have produced conflicting results. Some studies show that air pollutants are confounders and/or effect modifiers of the mortality-temperature association, while some other studies indicate that air pollutants have no significant impact on mortality with temperature. Studies conducted in the USA, for example, Bell *et al.* [50] and Zanobetti *et al.* [54] considered PM_10_ (as well as PM_2.5_ in Zanobetti *et al.*’s study) and O_3_, Basu *et al.* [55] considered PM_10_, PM_2.5_, O_3_, NO_2_, CO and SO_2_, found no significant confounding or effect modification by pollution on the association between temperature and mortality. Stafoggia *et al.* [56] also reported no confounding by O_3_ in Italy and Canada, respectively. However, PM_10_ was found to be confounder in Monterrey, Mexico [47], Sydney, Australia [57], and Brisbane, Australia [58]. Second, our study is limited to 26 regions in the south and west of China due to lack of data from other locations. Subsequent investigations including more regions are expected to increase our insight into the relationship between cardiovascular mortality and temperature in China.

## 5. Conclusions

In this study, we used statistical models to quantify the association between cardiovascular mortality and temperature for 26 regions in the south and west of China. Our results show that both cold and heat stressors have non-negligible public health effects on cardiovascular mortality [59,60], with variations observed between regions. In addition, although people may have developed certain biologic adaptations to their local climate, behavioral and technological adaptations are still needed [61].

This study proposed a Bayesian network as a new approach to learn the impact of social economic and demographic factors on cold-related and heat-related cardiovascular mortality. Compared with linear regression model, which is the most commonly used model in this kind of studies, Bayesian network has the advantage of integrate multiple factors within a consistent system, and modeling the mutual and hierarchal relationships of the variables without causing a problem of multicollinearity. More importantly, the method proposed in this study is general and can also be applied to other regions.

China is a very large country, with variations in socioeconomic and demographic conditions between cities. Thus, additional studies of different climates and socioeconomic and demographic conditions are needed, especially in the less developed regions. Moreover, these region-specific socioeconomic and demographic conditions should be taken into consideration for developing customized prevention and adaptation in public health programs for the regions vulnerable to high/low temperatures. For example, given the social economic and demographic conditions of a specific region, the probability of being at high risk of cold-related (or heat-related) cardiovascular mortality could be estimated. In addition, according to Table 3, women tend to have higher risk at high temperatures. When high weather conditions or heat weaves occur, preventive steps and developing public health programs should give more attention to women.

## Supplementary Files

Supplementary File 1
