# Supplementary material for: Cardiovascular Mortality Associated with Low and High Temperatures: Determinants of Inter-Region Vulnerability in China
Source: Int J Environ Res Public Health. 2015 May 27;12(6):5918–33. doi: 10.3390/ijerph120605918 (PMC4483679; doi:10.3390/ijerph120605918)
Supplement: Supplementary File 1 [file ijerph-12-05918-s001.pdf]

Cardiovascular Mortality Associated with Low and High Temperatures: Determinants of Inter-Region Vulnerability in China

Table S1. Reference values of low temperature and high temperature.

Region	Reference Value of Low Temperature	Reference Value of High Temperature	Region	Reference Value of Low Temperature	Reference Value of High Temperature
Chaohu City	2.41	28.27	Binyang County	11.13	29.17
Yushan District	2.36	28.28	Liubei District	9.69	30.25
Daguan District	3.39	29.16	Xiufeng District	7.89	29.44
Tianchang City	1.80	27.79	Hepu County	12.69	29.33
Mengcheng County	1.00	27.32	Lingyun County	10.82	27.73
Jing County	3.15	28.48	Luochengyaolaozu Autonomous County	9.32	29.04
Tianxin District	4.91	30.23	Meilan District	16.56	28.88
Liuyang City	4.64	29.38	Ding'an County	17.40	28.91
Pingjiang County	4.45	29.01	Chengguan District	0.20	17.57
Wuling District	4.94	29.30	Mozhugongka County	−1.34	15.56
Suxian District	5.56	30.42	Naidong County	0.42	16.57
Hongjiang City	4.82	27.93	Jiangzi County	−2.89	13.48
Fenghuang County	5.30	28.21	Milin County	1.12	16.49

Table S2. Splits of the factors for percent changes in cardiovascular mortality at low (PC_L) and high (PC_H) temperatures.

Quantitative factors	Discretization Intervals for PC_L	Discretization Intervals for PC_H
number of hospital beds per 10,000 people	[4.95, 21.11], (21.11, 109.38]	[4.95, 30.71], (30.71, 109.38]
per capita years of education	[5.18, 5.25], (5.25, 12.31]	[5.18,7.00], (7.00, 12.31]
%uneducated	[0.49, 1.07], (1.07, 31.93]	[0.49, 29.84], (29.84, 31.93]
%female	[46.66, 50.08], (50.08, 50.94]	[46.66, 50.83], (50.83, 50.94]
%urban_residents	[9.28, 92.75], (92.75, 99.42]	[9.28, 16.16], (16.16, 99.42]
%65+	[3.49, 5.65], (5.65, 12.68]	[3.49, 9.51], (9,51, 12.68]
%agriculture	[3.36, 5.77], (5.77, 89.48]	[3.36, 85.33], (85.33, 89.48]
%industry	[1.02, 7.08], (7.08, 51.24]	[1.02, 6.36], (6.36, 23.14], (23.14, 51.24]
%service	[9.50, 72.85], (72.85, 74.39]	[9.50, 10.90], (10.90, 74.39]

Notes: PC_L represents percent change in cardiovascular mortality at low temperature; PC_H represents percent change in cardiovascular mortality at high temperatures.

Table S3. Estimated percent change (%) in cardiovascular mortality at low (PC_L) and high temperatures (PC_H).

Region	PC_L(%)	PC_H(%)	Region	PC_L(%)	PC_H(%)
Chaohu City	2.45	7.81	Binyang County	0.81	5.13
Yushan District	1.19	8.29	Liubei District	0.00	10.58
Daguan District	0.95	5.01	Xiufeng District	0.07	10.29
Tianchang City	0.00	5.47	Hepu County	0.12	8.14
Mengcheng County	0.40	2.68	Lingyun County	0.32	8.66
Jing County	0.47	0.29	Luochengyaolaozu Autonomous County	0.66	0.00
Tianxin District	1.37	7.26	Meilan District	0.00	3.06
Liuyang City	0.97	9.27	Ding'an County	4.77	10.84
Pingjiang County	0.34	8.63	Chengguan District	1.32	0.00
Wuling District	0.76	10.48	Mozhugongka County	0.00	18.25
Suxian District	0.74	0.00	Naidong County	2.68	8.32
Hongjiang City	0.00	2.41	Jiangzi County	5.59	12.27
Fenghuang County	1.40	1.74	Milin County	1.05	57.39

Notes: PC_L represents percent change in cardiovascular mortality at low temperature; PC_H represents percent change in cardiovascular mortality at high temperatures.

© 2015 by the authors; licensee MDPI, Basel, Switzerland. This article is an open access article distributed under the terms and conditions of the Creative Commons Attribution license (http://creativecommons.org/licenses/by/4.0/).
